# Deleterious Metabolic Effects of High Fructose Intake: The Preventive Effect of *Lactobacillus kefiri* Administration

**DOI:** 10.3390/nu9050470

**Published:** 2017-05-17

**Authors:** María Guillermina Zubiría, Sabrina Eliana Gambaro, María Amanda Rey, Paula Carasi, María de los Ángeles Serradell, Andrés Giovambattista

**Affiliations:** 1 Neuroendocrinology Laboratory, Multidisciplinary Institute of Cellular Biology (IMBICE, CICPBA-CONICET-UNLP), 526 10 y 11, La Plata 1900, Argentina; gzubiria@imbice.gov.ar (M.G.Z.); sabrigambaro@gmail.com (S.E.G.); mamandarey@gmail.com (M.A.R.); 2 Biology Department, School of Exact Sciences, Universidad Nacional de La Plata, La Plata 1900, Argentina; 3 Cátedra de Microbiología, Departamento de Ciencias Biológicas, Facultad de Ciencias Exactas, Universidad Nacional de La Plata (UNLP), 47 y 115 s/n, La Plata 1900, Argentina; paulacarasi@gmail.com

**Keywords:** gut microbiota, fructose-rich diet, adipose tissue, probiotics

## Abstract

Modern lifestyle and diets have been associated with metabolic disorders and an imbalance in the normal gut microbiota. Probiotics are widely known for their health beneficial properties targeting the gut microbial ecosystem. The aim of our study was to evaluate the preventive effect of *Lactobacillus kefiri* (*L. kefiri*) administration in a fructose-rich diet (FRD) mice model. Mice were provided with tap water or fructose-added (20% *w*/*v*) drinking water supplemented or not with *L. kefiri*. Results showed that probiotic administration prevented weight gain and epidydimal adipose tissue (EAT) expansion, with partial reversion of the adipocyte hypertrophy developed by FRD. Moreover, the probiotic prevented the increase of plasma triglycerides and leptin, together with the liver triglyceride content. Leptin adipocyte secretion was also improved by *L. kefiri*, being able to respond to an insulin stimulus. Glucose intolerance was partially prevented by *L. kefiri* treatment (GTT) and local inflammation (TNFα; IL1β; IL6 and INFγ) was completely inhibited in EAT. *L. kefiri* supplementation generated an impact on gut microbiota composition, changing *Bacteroidetes* and *Firmicutes* profiles. Overall, our results indicate that the administration of probiotics prevents the deleterious effects of FRD intake and should therefore be promoted to improve metabolic disorders.

## 1. Introduction

Obesity has been defined by the World Health Organization as an Adipose Tissue (AT) excess that could be harmful to the organism, predisposing to pathologies such as type II Diabetes Mellitus, cardiovascular disease, dyslipidemias, fatty liver disease, and certain cancers. AT mass expansion is associated with serious changes in AT architecture and function, among which adipocyte hypertrophy is one of the most relevant features. Hypertrophic adipocytes are characterized by releasing high amounts of leptin, pro-inflammatory cytokines, low adiponectin, and insulin-resistance [1,2]. Furthermore, AT mass expansion induces a shift from an anti- to pro-inflammatory profile of immune cells resident in this tissue, leading to a general pro-inflammatory state of AT [3,4].

Obesity is a multifactorial disorder caused by the interaction of genetic background and environmental factors, such as altered eating habits [5]. Modern diets are characterized by high carbohydrate intake, especially fructose-sweetened beverages, and have been associated with high prevalence of overweight and Metabolic Syndrome (MS) in humans [6]. High-fructose feeding has been widely used in animal models to induce obesity and MS phenotype [7,8,9,10]. In the present study, we used a fructose-rich diet intake (FRD, 20% *w*/*v* in drinking water), which is far from fructose intake by humans, to generate a mice model of obesity. Previously, FRD has been related to the development of insulin resistance, dyslipidemias, increased abdominal AT mass, and changes in the pattern of AT adipokine secretion [7,8,11]. Partly, these metabolic disorders are a consequence of fructose-induced hepatic de novo lipogenesis, and the resulting increase in AT fatty acid uptake [12,13].

Gut microbiota is composed of 1 to 10 trillion microorganisms, mainly bacteria, among which approximately 90% belong to the *Bacteroidetes*, *Firmicutes*, *Actinobacteria* and *Proteobacteria* phyla [14]. Symbiotic relationships between bacteria and their hosts modulate several physiological processes such as nutrients uptake, metabolism, and immune response, among others [15]. Environmental factors such as diet, treatment with antibiotics, and exercise can modulate gut microbiota composition. Obesity has been associated with gut microbiota dysbiosis, contributing to the establishment of characteristic alterations related to obesity. Since transplantation of lean gut microbiota to obese mice can rescue the obese phenotype [16], strategies to manipulate the composition of the gut microbiota have gained considerable importance for metabolic pathologies management. Probiotics are defined as live microorganisms that, when administered, exert positive health effects in the host. It is largely accepted that probiotics are involved in the maintenance of healthy gut microbiota, and for this reason their use has emerged as a potential therapy against MS and obesity [17].

Kefir is a food product obtained by fermentation of milk with “kefir grains”. These grains are constituted by a complex symbiotic microbiota, mainly of yeast, lactic acid, and acetic acid bacteria confined in a matrix of polysaccharides and proteins [18,19]. Several health-promoting properties have been associated with kefir consumption [19,20], and the study of the beneficial properties of kefir-isolated microorganisms can be considered as a very important field for the development of functional foods. *Lactobacillus kefiri* is one of the most important lactobacilli obtained from kefir grains [21,22]. Different in vitro studies have revealed that secretion products and surface proteins from different *L. kefiri* strains can exert a protective action against intestinal pathogens such as *Salmonella enterica* [23] and *Clostridium difficile* [24]. Moreover, Carey and Kostrzynska reported that *L. kefiri* attenuates the pro-inflammatory response in intestinal epithelial cells induced by *Salmonella typhimurium*, and Hong et al. showed its influence on Th1 and pro-inflammatory cytokine production in macrophages [25,26]. 

Recent studies have demonstrated that *L. kefiri* CIDCA 8348 strain resists passage through simulated gastrointestinal conditions [27] and its oral administration is safe to mice [28]. Interestingly, mice treated with *L. kefiri* CIDCA 8348 showed a down-regulation of the gene expression of pro-inflammatory mediators and an up-regulation of anti-inflammatory molecules, secreted IgA and mucins in the gut [29]. Based on this evidence, we aimed to evaluate the preventive effect of *L. kefiri* CIDCA 8348 administration on the metabolic alterations caused by FRD in mice.

## 2. Material and Methods

### 2.1. Bacterial Strain and Growth Conditions

A kefir-isolated *Lactobacillus kefiri* strain (CIDCA 8348, *L. kefiri*) [30] from the collection of the “Centro de Investigación y Desarrollo en Criotecnológico de Alimentos” (La Plata, Argentina) was used for experiments. The strain was cultured in MRS-broth (DIFCO, Detroit, MI, USA) at 37 °C for 48 h under aerobic conditions. Frozen stock cultures were stored at −80 °C in skim milk until use.

### 2.2. Animals and Treatment

Normal adult male Swiss mice (four months of age, *n* = 15 mice per group) were kept in a temperature-controlled environment (20–22 °C and fixed 12 h light/12 h dark cycle, lights on at 07:00 a.m.) and fed *ad libitum* with Purina commercial rat chow. Mice were divided into two groups: one was provided with tap water and the other with a 20% fructose solution (*w*/*v*, Sigma-Aldrich, St. Louis, MO, USA) added to tap water for 6 weeks (conventionally called fructose rich diet, FRD). Each group was randomly divided and administered *L. kefiri* (10^8^ CFU dissolved in milk; CTR-Lk and DRF-Lk groups) or milk alone (CTR and FRD groups) by oral gavage every 48 h during the 6-week diet. Food intake and body weight were measured every 48 h. On experimental day, mice were euthanized under non-fasting conditions (between 08:00 a.m. and 09:00 a.m.) and trunk blood was collected; plasma samples were then frozen (−20 °C) until metabolite measurements (Section 2.3). Inguinal AT (IAT, subcutaneous depot), Epididymal AT (EAT, visceral depot) and Retroperitoneal AT (RPAT, visceral depot) were aseptically dissected and weighed. EAT was kept in sterile Dulbecco’s Modified Eagle’s Medium-Low Glucose (1 g/L) (DMEM-LG) for further procedures. Animals were euthanized according to protocols for animal use, in agreement with National Institutes of Health (NIH) guidelines for the care and use of experimental animals. All experiments were approved by our Institutional Animal Care Committee (approval code 020916).

### 2.3. Peripheral Metabolite Measurements

Non-fasting plasma levels of leptin (LEP, *n* = 15) were determined by specific radioimmunoassays (RIAs) previously developed in our laboratory [31]. Non-fasting plasma levels of glucose (Glu, *n* = 15) and triglycerides (Tg, *n* = 15) were measured using commercial kits (Wiener Lab., Rosario, Argentina).

### 2.4. EAT Adipocyte Isolation and Incubation

Fresh EAT pads were dissected, weighed and digested with collagenase as previously reported [32]. Briefly, fat tissue was minced and digested using 1 mg/mL collagenase solution in DMEM (at 37 °C, for 1 h). After centrifugation (1000 rpm for 15 min), floating mature adipocytes were separated and diluted up to a density of approximately 200,000 cells per 900 μL DMEM-1% BSA. Adipocytes were distributed in 24 multi-well plates and incubated for 45 min at 37 °C in a 5% CO_2_ atmosphere with medium alone (basal) or medium containing 10 nM insulin (Novo Nordisk Pharma AG, Küsnacht, Switzerland) [33]. After incubation, medium was carefully aspirated and kept frozen (−20 °C) until measurement of LEP concentrations as described above (*n* = 6 independent experiments).

### 2.5. EAT Pad Histology

For histological studies, freshly dissected EAT pads (*n* = 4 per group) were fixed in 4% paraformaldehyde, then washed with tap water, immersed in a series of graded ethanol solutions (70%, 96% and 100%), and clarified in xylene before paraffin embedding [34]. Four-micrometer sections were taken from different levels of the blocks and stained with hematoxylin-eosin. Quantitative morphometric analysis was performed using a RGB CCD Sony camera and Image Pro-Plus 4.0 software (Image ProPlus6.0, Rockville, MD, USA). For each tissue sample, seven sections and three levels were selected. Systematic random sampling was used to select 15 fields for each section (magnification, ×400) and 2500 cells per group were examined. Adipocyte area was measured.

### 2.6. Glucose Tolerance Test (GTT)

Four days before the end of the protocol, six mice from each experimental group were fasted for 10 h (from 10:00 p.m. to 8:00 a.m.) and then glucose (2 mg/kg BW) was administered via intraperitoneal (IP) injection. Blood was collected by the tail cut method. Glucose was measured at 0, 30, 60 and 120 min after glucose challenge by one-touch glucometer (Accu-Chek Performa, Roche, Mannheim, Germany). Area under the curve was calculated using Graph Pad Prism 6.0 (GraphPad Software Inc., San Diego, CA, USA).

### 2.7. Liver Lipid Content

Fifty mg of the liver (*n* = 6 per group) was homogenized in a 5% solution of 500 μL Triton X-100 in phosphate-buffered saline (PBS). The homogenate was incubated at 80–100 °C for 5 min and centrifuged at 10,000× *g* for 10 min. Triglyceride (Tg) was measured in the supernatants using a commercial kit (Wiener Lab, Rosario, Argentina).

### 2.8. RNA Isolation and Quantitative Real-Time PCR (qRT-PCR)

Total RNA from EAT (*n* = 6 per group) was isolated by the Trizol extraction method (Invitrogen, Life Tech., Carlsbad, CA, USA) and reverse-transcribed using random primers (250 ng) and RevertAid Reverse Transcriptase (200 U/μL, Thermo Scientific, Vilnius, Lithuania). Two μL cDNA were amplified with HOT FIRE Pol EvaGreenqPCR Mix Plus (Solis BioDyne, Tartu, Estonia) containing 0.5 μM of each specific primer, using a Rotor Gene Q (Qiagen, Hilden, Germany). Polymerase chain reaction (PCR) efficiency was near 1. Expression levels were analyzed for β-actin (ACTβ, reporter gene), Adiponectin (Adipo), Leptin (Ob), Lipoprotein Lipase (LPL), Fatty Acid Synthase (FAS), Hormone Sensitive Lipase (HSL), Adipose Triglyceride Lipase (ATGL), Tumor Necrosis Factor α (TNFα), Interleukin 1β (IL1β), Interleukin 6 (IL6), and Interferon γ (IFNγ). Designed primers are shown in Table 1. Relative changes in the expression level of one specific gene (ΔΔ*C*_t_) were calculated by the Δ*C*_t_ method.

### 2.9. Leptin Measurement

Medium LEP concentration was determined by specific RIA [35]. In this assay, the standard curve ranged between 50 and 12,500 pg/mL, with intra- and inter-assay variation coefficients of 4–6% and 5–8%, respectively.

### 2.10. Microbiota Analysis in Feces

Fecal samples were collected at the end of the experimental protocol and were stored at −80 °C prior to use for microbiota analysis. DNA extraction was performed using the *AccuPrep* Stool DNA Extraction Kit (Bioneer, Daejeon, Korea) according to the manufacturer’s instructions. 

#### 2.10.1. Quantitative PCR of Microbiota Populations

Quantification of bacterial populations was carried out using primers synthesized by Genbiotech (Buenos Aires, Argentina). Primer sequences were previously described [29,36]. PCR reactions were performed in a Rotor Gene Q (Qiagen, Hilden, Germany) using HOT FIRE Pol EvaGreenqPCR Mix Plus (Solis BioDyne, Tartu, Estonia). Twenty ng DNA and 0.2 μmol L^−1^ of each primer were used in PCR mix. A negative control reaction without template was included for each primer combination. Melting curve was conducted from 70 °C to 90 °C, read every 0.5 °C during 2 s. For standard curves we used PCR products generated from a pool of purified genomic DNA from the different samples and the primers previously described [29,36]. Results were expressed as number of copies/g wet weight feces.

#### 2.10.2. Qualitative Analysis by PCR-DGGE

Primers 518r (5′-ATTACCGCGGCTGCTGG-3′) and 338f (5′-CTCCTACGGGAGGCAGCAG-3′) coupled to a 50-GC clamp [37], targeting the V3 region of the 16S rRNA subunit [38], were used to assess microbial diversity in each sample. PCR was performed in a Stratagene Gradient Cycler (Agilent Technologies Inc., Philadelphia, PA, USA) using 1U of PFU DNA Polymerase (PB-L, EmbioTec SRL, Buenos Aires, Argentina) per 50 pg of DNA template. The PCR products were separated in 8% polyacrylamide gels (37.5:1 acrylamide:bisacrylamide) with a range of 40–60% denaturing gradient (100% denaturant consisted of 7 M urea and 40% deionized formamide) cast in a DDGE-2401 device (C.B.S Scientific Co., Del Mar, CA, USA). The electrophoresis was performed in TAE 0.5X buffer for 16 h at a constant voltage of 100 v and a temperature of 60 °C. Gels were stained with SYBR Gold 0.01 μL/mL (Invitrogen, Life Technologies, Carlsbad, CA, USA) in TAE 1X buffer and visualized in a Bio-Rad Universal Hood II gel documentation system (Bio-Rad laboratories Inc., Hercules, CA, USA). PyElph 1.4 software was used to calculate the dendrograms using the UPGMA (unweighted pair group method with arithmetic mean clustering algorithm) [39].

### 2.11. Statistical Analysis

Results are expressed as mean values ± S.E.M. Data were analyzed by ANOVA (one-way) method followed by Fisher´s test. Body weight data were analyzed using a multivariate test (IBM SPSS statistics 22, IBM Corp., New York, NY, USA). To determine the differential effect of the treatments, ANOVA (two-way) analysis was performed followed by Tukey’s test. *p* values lower than 0.05 were considered statistically significant. All statistical tests were performed using GraphPad Prism 6.0 (GraphPad Software Inc., San Diego, CA, USA).

## 3. Results

### 3.1. L. kefiri Administration Prevents Body Weight Gain and AT Expansion

**Figure 1:**
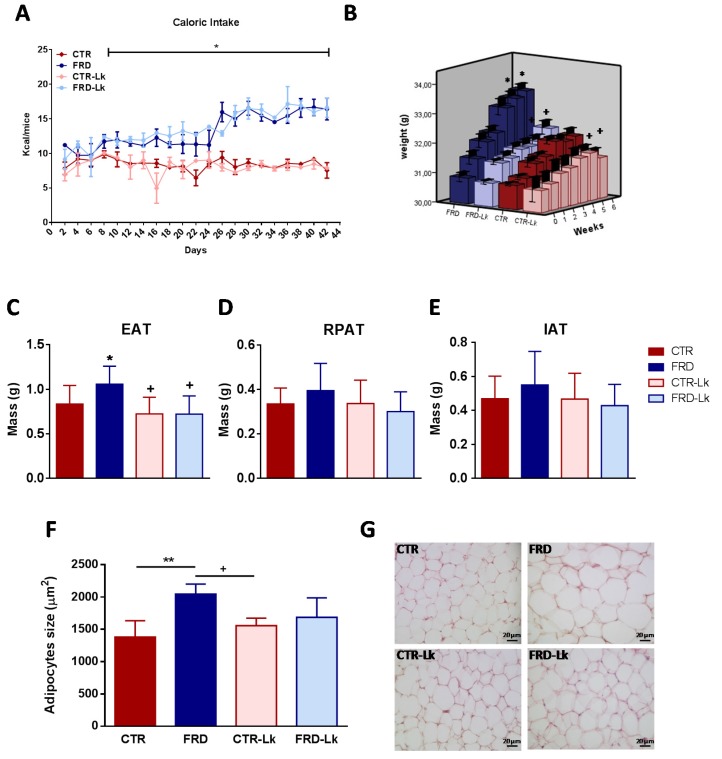
Mean of caloric intake, body weight and AT expansion. (**A**) Caloric intake (* *P* < 0.05 vs. CTR and CTR-Lk) and (**B**) body weight from the different groups. (* *P* < 0.05 vs. CTR. ^+^ *P* < 0.05 vs. FRD) (**C**) EAT, (**D**) RPAT and (**E**) IAT mass were measured. * *P* < 0.05 vs. CTR and ^+^ *P* < 0.05 vs. FRD. (*n* = 15 mice per group). (**F**) EAT adipocyte size. (*n* = 4 mice per group). ** *P* < 0.001 vs. CTR and ^+^ *P* < 0.05 vs. FRD. (**G**) Representative EAT histological samples stained with hematoxylin-eosin. Values are means ± SEM.

Caloric intake for FRD mice was higher than that for CTR mice (Figure 1A; *P* < 0.05) and, as expected, was accompanied by an increase in body weight (Figure 1B; *P* < 0.05 vs. CTR). *L. kefiri* administration to CTR mice (CTR-Lk group) did not modify caloric intake or body weight compared to CTR animals. However, administration of *L. kefiri* during FRD intake prevented the increase in body weight without changing the caloric intake (*P* < 0.05 vs. FRD). Multivariate analysis showed an interaction between diet and probiotic administration had a significant effect (*P* = 0.02). FRD consumption has been widely used in animal models to induce metabolic disorders as those observed in human MS; one of these features is the visceral AT expansion. Our results showed that FRD induced a significant increase in EAT mass (*P* < 0.05). Interestingly, probiotic treatment inhibited EAT mass expansion induced by FRD intake (FRD-Lk mice; *P* < 0.05) and also decreased EAT mass in CTR-Lk mice compared to CTR (Figure 1C; *P* < 0.05). With regard to other AT depots studied, although no significant differences were found among experimental groups, FRD mice showed a trend toward increased IAT and RPAT mass, which was not observed in FRD-Lk mice (Figure 1D,E).

Unhealthy AT expansion has been associated with hypertrophic adipocytes while hyperplastic AT expansion prevents AT dysfunctions. Histological analysis of adipocyte size from EAT showed that *L. kefiri* administration did not affect cell size in CTR-Lk mice (Figure 1F). On the other hand, FRD induced an increase in adipocyte size (*P* < 0.01) that was not observed in FRD-Lk adipocytes. Thus, high fructose consumption generated an unhealthy EAT expansion but was partially prevented by the probiotic treatment.

### 3.2. Metabolic Alterations and Glucose Homeostasis Impairment Were Improved by Probiotic Treatment

**Figure 2:**
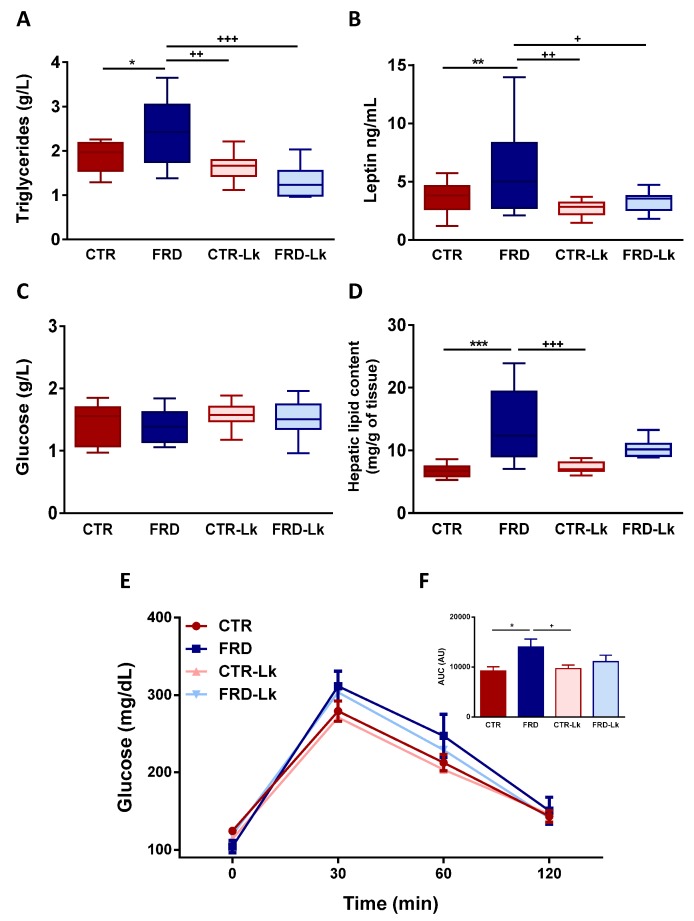
Metabolic Parameters and Glucose Tolerance Test. (**A**) Triglycerides, (**B**) Leptin and (**C**) Glucose plasma concentrations were recorded. (*n* = 15 mice per group). (**D**) Liver triglycerides content was assessed in the four groups. Values are means ± range (*n* = 6 mice per group). (**E**) GTT was performed four days before to the end of the protocol. Glucose concentration was measured at 0, 30, 60 and 120 min after glucose challenge. (**F**) Area under the curve was calculated. Values are means ± SEM (*n* = 6 mice per group). * *P* < 0.05; ** *P* < 0.01 and *** *P* < 0.001 vs. CTR. ^+^ *P* < 0.05; ^++^ *P* < 0.01 and ^+++^ *P* < 0.001 vs. FRD.

FRD mice showed an impaired metabolic profile, characterized by higher plasmatic concentration of Tg and LEP than CTR mice, without changes in Glu plasmatic levels (Figure 2A–C; *P* < 0.05 and *P* < 0.01, respectively). *L. kefiri* treatment did not modify these parameters in CTR-Lk mice. Interestingly, FRD-Lk mice showed circulating levels of Tg and LEP similar to CTR mice, which reveals the beneficial effect of *L. kefiri* administration on the metabolic profile from high-fructose feeding mice.

Additionally, liver lipid content was similar for CTR and CTR-Lk mice (Figure 2D). As expected, considering the strong lipogenic capacity of fructose, FRD mice showed higher liver lipid levels (*P* < 0.05). This increase was in part attenuated by the administration of *L. kefiri* (FRD-Lk group).

GTT was performed to assess glucose homeostasis in the different groups. Administration of *L. kefiri* to CTR mice did not modify GTT response, as shown in Figure 2E. For the case of FRD group, we observed an impaired glucose tolerance, which was clearly evidenced in a higher area under the curve (Figure 2F; *P* < 0.05). This glucose intolerance was partially prevented by *L. kefiri* treatment (FRD-Lk).

### 3.3. L. kefiri Administration Reduces EAT Dysfunctions Induced by FRD

**Figure 3:**
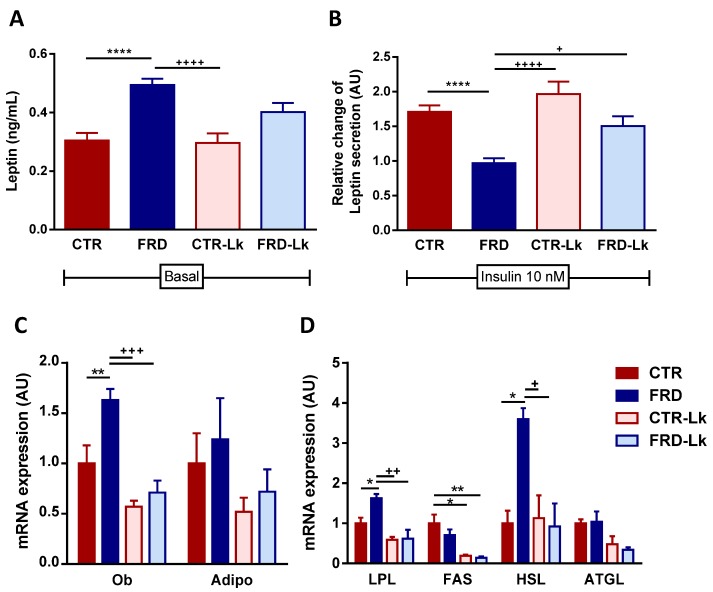
Improvement of EAT function. (**A**) Leptin secretion from cultured adipocytes in basal conditions. (**B**) Relative change of leptin secretion after insulin stimulus compared to basal secretion for each group. (*n* = 6 independent experiments). EAT mRNA expression of (**C**) LEP (Ob) and adiponectin (Adipo) and (**D**) lipid metabolism-related enzymes (LPL, HSL, ATGL, FAS). (*n* = 6 mice per group). Values are means ± SEM. * *P* < 0.05; ** *P* < 0.01 and **** *P* < 0.0001 vs. CTR. ^+^ *P* < 0.05; ^++^ *P* < 0.01; ^+++^ *P* < 0.001 and ^++++^ *P* < 0.0001 vs. FRD.

AT dysfunction directly correlates with adipocyte size. Hypertrophic adipocytes are insulin resistant and secrete an altered adipokyne pattern (LEP and Adipo) and pro-inflammatory cytokines. As shown in Figure 3A,B, isolated EAT adipocytes from CTR-Lk secreted similar amount of LEP as CTR mice, both spontaneously and after insulin stimulation. FRD hypertrophic adipocytes secreted more LEP than CTR ones, and did not respond to insulin stimulus (*P* < 0.0001 and *P* < 0.0001 vs. CTR, respectively). Under basal condition, adipocytes from FRD-Lk showed an intermediate secretion of LEP between CTR and FRD adipocytes, suggesting a partial protection exerted by *L. kefiri* administration on the impairment of LEP secretion caused by FRD. Interestingly, when FRD-Lk adipocytes were insulin-stimulated, they significantly increased their LEP release (*P* < 0.001 vs. FRD-Lk basal), indicating insulin sensitivity recovery (*P* < 0.05 vs. FRD).

When mRNA expression was analyzed, EAT from FRD expressed significantly higher LEP levels than CTR adipocytes (Figure 3C; *P* < 0.01), while *L. kefiri* administration protected FRD-Lk mice from this increase (Figure 3C; *P* < 0.001 vs. FRD), in agreement with results detailed above. Neither FRD nor *L. kefiri* treatment altered adiponectin mRNA levels. To assess the status of lipid metabolism in EAT we evaluated the expression of different enzymes involved in lipolysis/lipogenesis pathway. As shown in Figure 3D, EAT from FRD mice expressed higher mRNA levels of HSL and LPL than CTR (HSL: *P* < 0.05 and LPL: *P* < 0.05) and CTR-Lk mice (HSL: *P* < 0.05 and LPL: *P* < 0.01), whereas Lk administration to FRD mice prevented this increase (HSL: *P* < 0.05 and LPL: *P* < 0.01 vs. FRD).

### 3.4. L. kefiri Treatment Protects EAT from Inflammation Induced by FRD

**Figure 4:**
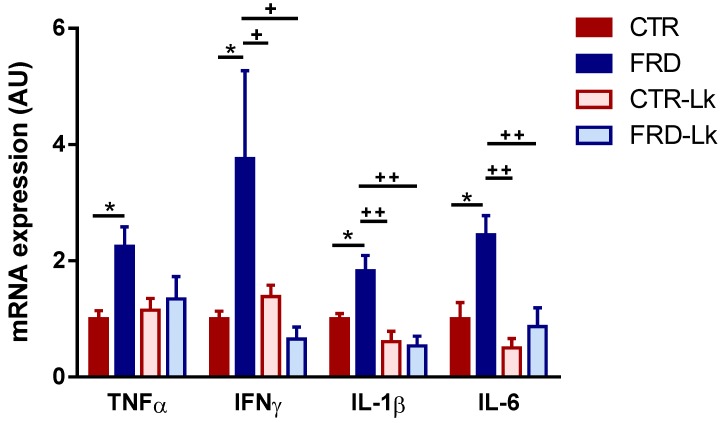
Inflammatory profile of EAT. Gene expression of pro-inflammatory cytokines (TNFα, INFγ, IL1β, IL6) in EAT from the four experimental groups. Values are means ± SEM (*n* = 6 mice per group). * *P* < 0.05 vs. CTR. ^+^ *P* < 0.05 and ^++^ *P* < 0.01 vs. FRD.

It is well known that obesity is associated with AT chronic inflammatory state caused by the change of AT resident immune cells from anti-inflammatory Type 2 to pro-inflammatory Type 1, favoring insulin-resistance. In this regard, our results showed that FRD intake induced an inflammatory state in EAT, evidenced by significant increase of IL6, IL1β, TNFα and IFNγ expression (Figure 4; *P* < 0.05 vs. CTR), that was prevented by *L. kefiri* treatment (IFNγ: *P* < 0.05; IL1β: *P* < 0.01 and IL6: *P* < 0.01 vs. FRD). Noticeably, we found that *L. kefiri* administration did not modify mRNA levels of pro-inflammatory cytokines in EAT from CTR-Lk mice.

### 3.5. Effects of FRD and L. kefiri Administration on Gut Microbiota Composition

**Figure 5:**
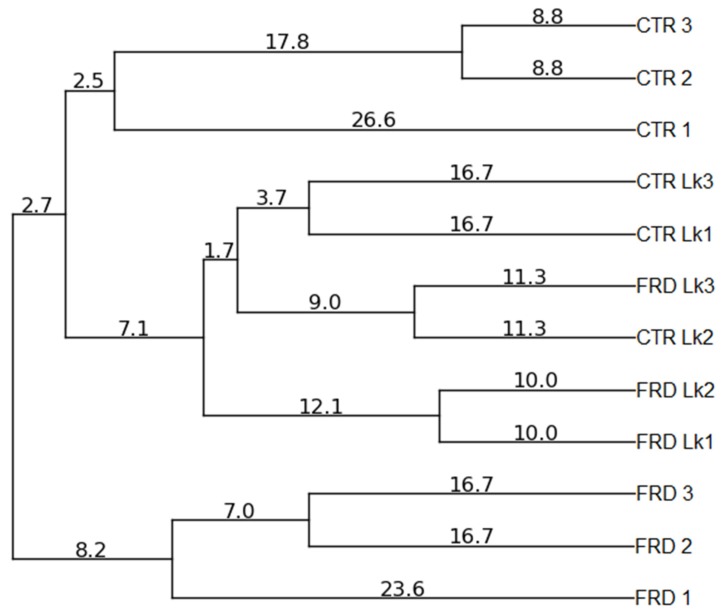
Qualitative analysis of fecal microbiota profile. Dendrogram for the total bacterial DGGE profiles. Clustering analysis was performed using the UPGMA linkage.

**Figure 6:**
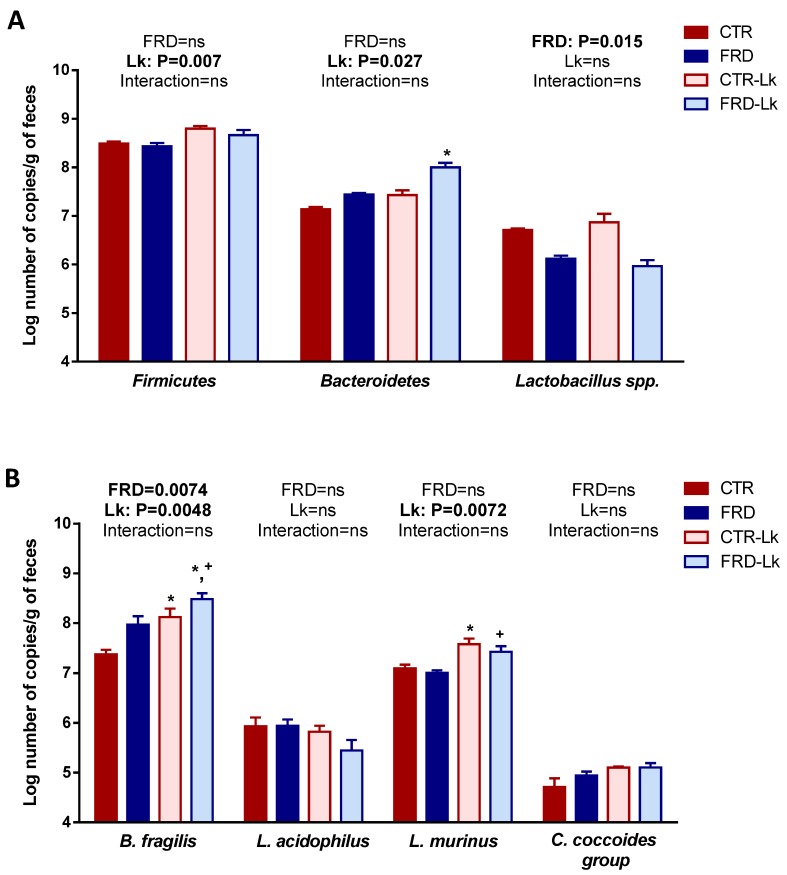
Analysis of microbiota composition in feces. (**A**) Lactobacillus spp., Lactobacillus acidophilus (*L. acidophilus*) and Lactobacillus murinus (*L. murinus*) quantification. (**B**) Firmicutes and Bacteroidetes phyla, Clostridium coccoides group (*C. coccoides* group) and Bacteroides fragilis (*B. fragilis*) quantification. Two-way ANOVA was performed for variable analysis and Tukey’s multiple comparisons post-test was performed for group-to-group comparisons. ns = no significant differences. Values are means ± SEM (*n* = 3 mice per group). * *P* < 0.05 vs. CTR, ^+^ *P* < 0.05 vs. FRD.

To study the effects of diet and probiotic administration on gut microbiota structure, we analyzed the fecal bacterial composition by PCR-DGGE and qPCR. Both FRD and Lk administration produced qualitative changes in the microbial community composition, since the cluster analysis based on the Pearson product-moment correlation coefficient and UPGMA linkage allowed differentiation of the experimental groups in separated clusters (Figure 5), without differences in the number of amplification bands generated from each sample (not shown). Regarding qPCR assays, we performed two-way ANOVA analysis to determine if FRD or Lk or the interaction of both variables have some effect on fecal bacterial amounts. Firstly, no differences in the total number of bacteria between groups were found, although a trend to increase was observed in mice under probiotic treatment, independently of diet intake (Table 2). When specific bacterial phyla were analyzed, we found a different effect regarding diet and probiotic administration. FRD feeding decreased the *Lactobacillus* spp. (Figure 6A, *P* = 0.015) and increased *Bacteroides fragilis* quantities independently of probiotic administration (Figure 6B, *P* = 0.0074). On the other hand, when *L. kefiri* administration was analyzed we found an increase in *Firmicutes* and *Bacteroidetes* phyla (Figure 6A, *P* = 0.007 and *P* = 0.027 respectively), and in *Lactobacillus murinus* and *Bacteroides fragilis* species (Figure 6B, *P* = 0.0048 and *P* = 0.0072 respectively), in spite of mice receiving or not the FRD. No differences were found in *L. acidophilus* group when both variables were analyzed.

After the overall variable analyses, we compared the microbiota composition among the different groups. Interestingly, we found that *Bacteroidetes* population from FRD-Lk mice was significantly increased compared to CTR (*P* < 0.05). Accordingly, *Bacteroides fragilis* (*Bacteroidetes* phylum) was also increased in FRD-Lk (*P* < 0.05 vs. CTR and FRD) and in CTR-Lk mice (*P* < 0.05 vs. CTR). Additionally, *Lactobacillus murinus* was also more abundant in FRD-Lk and in CTR-Lk (*P* < 0.05) than their counterparts without probiotic administration (Figure 6B). These changes in the microbiota composition in Lk-treated mice could suggest the presence of a healthier microbiota, which could be related to the beneficial metabolic changes found in them.

## 4. Discussion

Over the few last decades, the importance of the symbiotic relationship between gut microbiota and host in energy absorption, immune system and metabolism has been described [40]. Alterations caused by environmental factors on gut microbiota composition could lead to host metabolic disorders, as has been observed in both obese humans and rodents [41,42,43]. Since probiotics modulate gut microbiota and also affect host metabolism, the use of probiotics has been associated with several metabolic improvements in obese phenotypes [40,44]. In this regard, several studies have demonstrated the benefits of the use of lactobacilli as probiotics, improving liver pathologies, among others [45,46].

Fructose-sweetened beverages are one of the most remarkable components of modern diets and their consumption has increased notably in the last few decades [6]. Although diet is one factor that can affect gut bacterial profile in early life, as well as in adulthood, the effect of fructose on gut microbiota has been poorly studied. In fact, the use of probiotics as a preventive tool has been addressed mostly in high-fat diet models. FRD has been widely used to induce MS and obesity in animal models [7,8,11]. Our current results show that FRD intake for six weeks was effective in inducing an increase in body weight and EAT mass. These changes were accompanied by higher Tg and LEP plasma levels, peripheral insulin-resistance and increased liver lipid content, confirming the deleterious effects caused by this diet.

In the present study, we proposed to evaluate the potential protective effect of *L. kefiri* against the metabolic disorders induced by FRD. *L. kefiri* is a microorganism derived from kefir grains. Previous reports have demonstrated that kefir improves fatty liver syndrome in *ob*/*ob* mice [47] and metabolic parameters in spontaneously hypertensive rats [48]. Interestingly, in our model the administration of *L. kefiri* completely prevented the alterations caused by FRD intake, which strongly support the beneficial effect of this probiotic. In concordance with our results, other studies have shown that the use of *Lactobacillus* species as probiotics improves the metabolic disorders induced by the FRD [49,50].

There is growing evidence that AT-gut microbiota axis modulates several metabolic processes, including adipokine secretion and lipid metabolism, among others [51]. For this reason, the maintenance of healthier gut microbiota is relevant for the normal function of the AT. It was demonstrated that probiotic administration to high-fat fed mice reduced the infiltration of pro-inflammatory macrophages into AT and also adipocyte size [52]. In line with these findings, our results showed that *L. kefiri* administration to FRD-fed mice decreased the expression of several pro-inflammatory cytokines in EAT, indicating a prevention of the local pro-inflammatory state caused by FRD intake. Previously, it was demonstrated that orally administered *L. kefiri* induces an anti-inflammatory response in the gut of healthy mice [29]. This anti-inflammatory action could lead to the prevention of body weight gain and visceral fat accumulation, as proposed as an explanation for weight modification induced by other lactobacilli [53]. Furthermore, the anti-inflammatory effect was accompanied by a partial attenuation of adipocyte hypertrophy and an improvement of insulin-sensitivity in FRD-Lk adipocytes. It is largely accepted that adipocyte size is directly correlated with LEP secretion [1]. Moreover, LEP induces the secretion of pro-inflammatory cytokines, which in turn have a positive feedback to LEP [43]. It has been reported that periodic administration of probiotic mixture to obese MSG (monosodium glutamate) rats increased adiponectin levels and decreased the leptin concentration in AT and the visceral AT mass [54]. We observed that in vitro FRD-Lk adipocytes secreted an intermediate amount of LEP between CTR and FRD adipocytes, which was in accordance with the partial recovery of adipocyte size in FRD-Lk and the decrease in inflammation. Additionally, expression analysis in EAT from FRD mice that received *L. kefiri* showed lower levels of Ob, but no changes in Adipo mRNA levels compared to EAT from FRD mice. Several studies have demonstrated the beneficial effect of probiotic on lipid metabolism, by regulating the expression of lipid metabolism-related enzymes [55,56,57]. Specifically, a direct effect on AT lipid metabolism has been observed [58]. Our findings indicate that FRD intake generated an imbalance in the expression of lipogenic/lipolitic enzymes in EAT, that was recovered by *L. kefiri* administration. Overall, our results strongly support that *L. kefiri* treatment has several beneficial effects in AT metabolism and function, suggesting a tight communication between AT and the gut microbiota. However, further studies are needed to elucidate the mechanisms involved.

Different mediators have been proposed as a link between intestinal microbiota and host metabolism. One of these is short-chain fatty acids (SCFAs), mainly acetate, propionate and butyrate, which are generated as a result of bacterial fermentative metabolism. Due to its heterofermentative metabolism, *L. kefiri* produces lactic acid, ethanol and carbon dioxide as main metabolites of sugar fermentation [27]. To our knowledge, there are no scientific reports about SCFA production by *L. kefiri* strains. However, it is very interesting that Iraporda and coworkers recently demonstrated that lactate inhibits the activation of intestinal epithelial cells triggered by different pro-inflammatory stimuli [59,60]. SCFAs have been shown to generate protection against diet-induced obesity [61,62]. Several of their actions are mediated through activation of free fatty acid receptors (FFARs) [63], some of which are abundantly expressed in AT and may be involved in regulating lipid metabolism and glucose homeostasis [64]. Although we did not analyze the presence of lactic acid or SCFAs in fecal samples in our study, we cannot discard the hypothesis that the production of lactate or modifications in SCFA production may contribute to the preventive effect exerted by *L. kefiri* in our model. On the other hand, some trials suggest that another possible mechanism is a lower expression of the tight junction proteins that generates an increased gut permeability to lipopolysaccharides (LPS). These bacterial ligands can stimulate immune cells, such as those from AT, thus contributing to establish a chronic inflammatory state in obese individuals [41]. In fact, LPS plasma concentrations are increased in obese individuals [65], suggesting its contribution to endotoxemia and AT inflammation development during obesity.

Changes in diversity and number of bacteria in the intestinal microbiota during obesity has been proved, however, no consensus has been reached about the composition of a healthy or unhealthy gut microbiome [66]. In our studies, the PCR-DGGE assay showed that both FRD and Lk administration produced qualitative changes in the microbial community composition, while no changes in diversity were observed. Studies in animal models have shown many controversies related to *Firmicutes* and *Bacteroidetes* abundance, depending on the diet and length of treatment (high fat, high carbohydrate or high fiber diets). Some studies showed that fructose intake produces a decrease in *Bacteroidetes*, while others stated no changes or even an increment [49,67]. Similarly, for *Firmicutes*, an increase or no changes were reported [49,50,67]. In our study, we analyzed if both variables studied (FRD and Lk administration) affected the microbiota composition of treated mice. Firstly, we did not find any changes in *Firmicutes* and *Bacteroidetes* phyla when FRD intake was analyzed. However, we did observe a decrease in fecal *Lactobacillus* spp. in FRD-mice, independently if they received or not *L. kefiri*. This result agrees with those previously shown by Di Luccia *et al.* and Jena *et al.*, who reported a decrease in *Lactobacillus* in high fructose-fed rats [67,68]. When we evaluated the effect of *L. kefiri* administration we observed that it has a significant positive influence in both *Firmicutes* and *Bacteroidetes* phyla, and also in two of the four specific populations studied, *B. fragilis* and *L. murinus*. All these changes evidence an effect per se of the *L. kefiri* strain, independent from the diet, and could be suggesting the establishment of a healthier bacterial community.

As mentioned before, most of the studies evaluating the use of probiotics have been performed in high-fat diet models. However, some works have studied the use of probiotics in high-fructose consumption models and its relationship with changes in microbiota composition. One report showed an increase in both *Firmicutes* and *Bacteroidetes* quantities in small intestine when FRD was co-administered with *L. rhamnosus* GG [49]. In our work, when compared group to group, a significant increase in *Bacteroidetes* was observed in FRD-Lk mice. On the other hand, Zhang et al. reported that FRD supplemented with *L. casei*, increased intestinal *Bacteroides fragilis* (*Bacteroidetes* phylum) and decreased *Clostridium* spp. (*Firmicutes* phylum) quantities, at the same time that it improved the oral glucose tolerance test in FRD-fed rats [50]. In line with these results, FRD-fed mice supplemented with *L. kefiri* showed an increase in *B. fragilis*, but no changes in *Clostridium coccoides* group, accompanied by an improvement of several metabolic alterations caused by FRD intake. Some reports have described beneficial effects of *B. fragilis*, including preventive effects against colitis and intestinal inflammation [69,70], and improvement in plasma levels of triglycerides and glucose [71]. Furthermore, we found that *L. murinus* population was higher in FRD-Lk and CTR-Lk mice. Previously, an increase of *L. murinus* in colon was associated with an improvement in the intestinal immunity [72]. Finally, when we analyzed *Lactobacillus* spp., we found a trend toward increase in CTR-Lk mice, similarly to the significant increase previously reported by our group [29]. It is worth to note that some of these results do not completely agree with Carasi et al. (2015) [29], which could be mostly related to the difference in the length of *L. kefiri* treatment (3 weeks vs. 6 weeks). Overall, these changes in microbiota populations may explain in part the anti-inflammatory and metabolic improvements generated by *L. kefiri* in Lk-treated mice. However, further studies are needed to determine the association between changes in microbiota composition, caused by *L. kefiri* administration, and the metabolic improvements found in our model.

## 5. Conclusions

In summary, this work showed, as expected, that a fructose-rich diet induced endocrine-metabolic alterations in mice that resemble those found in human MS. These alterations could be partially caused by a dysbiosis induced by FRD; however, more studies about effects of fructose intake on gut microbiota are needed. In our experimental model, we evidenced that FRD does not alter the *Bacteroidetes* and *Firmicutes* phyla, but decreases *Lactobacillus* spp. Moreover, we demonstrated the beneficial effects of *L. kefiri* as a probiotic, such as changing gut microbiota composition and preventing metabolic alterations and AT dysfunction induced by FRD. In this regard, we previously showed the anti-inflammatory action of *L. kefiri*, accordingly with a novel protective effect against AT inflammation. Finally, we have contributed to reinforce the importance of probiotics as a preventive treatment for metabolic alterations associated with obesity. In particular, the *L. kefiri* strain isolated from a natural food, such as kefir grains, emerges as a potential tool for obesity management.

## Figures and Tables

**Table 1:** Primers used for real time PCR analysis.

Gene	Sequence (5′-3′)	GBAN	Size Product (bp)
ACTβ	Fw: TTTGCAGCTCCTTCGTTGCC Rv: ACCCATTCCCACCATCACAC	NM_007393.5	189
Ob	Fw: ACCAGGATCAATGACATTTCACAC Rv: GGCTGGTGAGGACCTGTTGA	NM_008493.3	148
Adipo	Fw: GGAACTTGTGCAGGTTGGATG Rv: CCCTTCAGCTCCTGTCATTCC	NM_009605.5	171
LPL	Fw: AGGACCCCTGAAGACAC Rv: GGCACCCAACTCTCATA	NM_008509.2	149
ATGL	Fw: CCACTCACATCTACGGAGCC Rv: AATCAGCAGGCAGGGTCTTC	NM_001163689.1	198
HSL	Fw: AGTTACCATCTCACCTCC Rv: CTTGCTGTCCTGTCCTTC	NM_010719.5	94
FAS	Fw: CAAGCAGGCACACACAATGG Rv: GCCTCGGAACCACTCACA	NM_007988.3	141
TNFα	Fw: CATCTTCTCAAAATTCGAGTGACAA Rv: CCTCCACTTGGTGGTTTGCT	NM_013693.3	63
IFNγ	Fw: TGGCATAGATGTGGAAGAAAAGAG Rv: TGCAGGATTTTCATGTCACCAT	NM_008337.4	81
IL1β	Fw: CTTGTGCAAGTGTCTGAA Rv: AGGTCAAAGGTTTGGAAG	NM_008361.4	143
IL6	Fw: GTTCTCTG GAAATCGTGGAAA Rv: AAGTGCATCATCGTTGTTCATACA	NM_031168.2	77

Specific primers used for real time PCR analyses; Fw: Forward, Rv: Reverse; GBAN: GenBank Accession Number; bp: base pairs.

**Table 2:** Total bacteria quantification in feces.

	CTR	FRD	CTR-Lk	FRD-Lk
**Total Bacteria (*N* of copies/g of feces)**	3.73 × 10^9^ ± 5.6 × 10^8^	4.97 × 10^9^ ± 5.0 × 10^8^	5.94 × 10^9^ ± 6.7 × 10^8^	6.97 × 10^9^ ± 7.8 × 10^8^
